# The Effects of Di-(2-ethylhexyl)-phthalate Exposure on Fertilization and Embryonic Development *In Vitro* and Testicular Genomic Mutation *In Vivo*

**DOI:** 10.1371/journal.pone.0050465

**Published:** 2012-11-30

**Authors:** Xue-Feng Huang, Yan Li, Yi-Hua Gu, Miao Liu, Yan Xu, Yao Yuan, Fei Sun, Hui-Qin Zhang, Hui-Juan Shi

**Affiliations:** 1 Shanghai Medical College, Fudan University, Shanghai, China; 2 Reproductive Medical Center, The First Affiliated Hospital of Wenzhou Medical College, Wenzhou, China; 3 National Population and Family Planning Key Laboratory of Contraceptive Drugs and Devices, Shanghai Institute of Planned Parenthood Research, Shanghai, China; 4 Laboratory for Reproductive Biology, School of Life Science, University of Science and Technology of China, Hefei, China; University Hospital of Münster, Germany

## Abstract

The present study was undertaken to determine the reproductive hazards of Di-(2-ethylhexyl)-phthalate (DEHP) on mouse spermatozoa and embryos *in vitro* and genomic changes *in vivo*. Direct low-level DEHP exposure (1 μg/ml) on spermatozoa and embryos was investigated by *in vitro* fertilization (IVF) process, culture of preimplanted embryos in DEHP-supplemented medium and embryo transfer to achieve full term development. Big Blue® transgenic mouse model was employed to evaluate the mutagenesis of testicular genome with *in vivo* exposure concentration of DEHP (500 mg/kg/day). Generally, DEHP-treated spermatozoa (1 μg/ml, 30 min) presented reduced fertilization ability (*P*<0.05) and the resultant embryos had decreased developmental potential compared to DMSO controls (*P*<0.05). Meanwhile, the transferred 2-cell stage embryos derived from treated spermatozoa also exhibited decreased birth rate than that of control (*P*<0.05). When fertilized oocytes or 2-cell stage embryos were recovered by *in vivo* fertilization (without treatment) and then exposed to DEHP, the subsequent development proceed to blastocysts was different, fertilized oocytes were significantly affected (*P*<0.05) whereas developmental progression of 2-cell stage embryos was similar to controls (*P*>0.05). Testes of the Big Blue® transgenic mice treated with DEHP for 4 weeks indicated an approximately 3-fold increase in genomic DNA mutation frequency compared with controls (*P*<0.05). These findings unveiled the hazardous effects of direct low-level exposure of DEHP on spermatozoa's fertilization ability as well as embryonic development, and proved that *in vivo* DEHP exposure posed mutagenic risks in the reproductive organ – at least in testes, are of great concern to human male reproductive health.

## Introduction

Di-(2-ethylhexyl)-phthalate (DEHP), one of the endocrine disruptors (EDs), affects humans greatly in industry and commerce due to its extensive use in manufacture of plastic and non-polymer materials like lacquers, paints, adhesives, fillers and printing inks and cosmetics [1], [2]. Humans are exposed through inhalation, ingestion and dermal absorption on a daily basis. As a result, DEHP poses significant public health hazard. In recent years, the ubiquitous use of DEHP has drawn a lot of concern due to its adverse effects on reproductive system. Epidemiological studies indicated that DEHP exposure could be possibly toxic to human male reproduction. A significant correlation exists between DEHP and human sperm motility both in *in vitro* and *in vivo* conditions and DEHP results in a concentration- and duration-dependent decrease in sperm viability [3]. A study of male workers in polyvinyl chloride (PVC) plants even found adverse associations between DEHP concentration in ambient air and sperm motility as well as chromatin DNA integrity [4]. In animal experiments, DEHP has been evaluated for developmental and reproductive toxicity for several decades. DEHP administration throughout gestation in CD-1 mice resulted in an increased incidence of malformations and produced maternal and other embryofetal toxicity under some dose levels [5]. Other researchers observed that both male and female mice dosed prior to and during cohabitation period, would cause dose-dependent decreases in fertility and in the number and the proportion of pups born alive [6]. Nevertheless, amidst the accumulating evidence in support of an association between DEHP exposure and harmful reproductive effects [5]–[8], most researches focus on DEHP exposure via inhalation, ingestion or oral administration wherein only a vague concentration reached target organ or area. Although the cytotoxicity and genotoxicity potential of DEHP and its metabolites were found in *in vitro* experiments by decreasing cell viability and steroidogenic potential in mouse [9], [10], inducing DNA damage, altering mitotic rate, apoptosis, cell proliferation and activating a number of nuclear receptors in human cells or tissues [11], it remains unable to differentiate between direct effect of DEHP on germ cells or indirect effect of DEHP via byproducts generated by the liver like mono-2-ethylhexyl phthalate (MEHP) or indirect effect of DEHP mediated by the endometrium. So far, direct DEHP exposure on spermatozoa, especially its effects on fertilization and embryo developmental potential, has been rarely investigated. Parameters such as motility, viability and sperm DNA integrity are commonly used to estimate sperm quality, however, the fertilization capacity as well as subsequent embryonic development should be a more sensible and reliable indicators to evaluate sperm function. *In vitro* fertilization (IVF) procedure, has been undertaken not only in clinic practice for infertility treatment, but in toxicology for studying environmental toxicant hazards for gametes through fecundability, and it has been indicative of a decreased sperm quality in infertile couples subject to DEHP exposure [4], [12]. Conducted *in vitro*, a well-designed IVF assay could simplify the complicated physiology environment *in vivo*, and determine a single chemical's toxicity at a specific stage of fertilization. To our knowledge, there has been no study conducted on mouse spermatozoa with direct DEHP exposure, in this study, we utilized IVF as an assay to determine the effects of direct DEHP exposure at a low dose (1 μg/ml, 30 mins) on the fertilization potential of spermatozoa and early development of the resulting embryos. Parts of 2-cell stage embryos were also transferred into recipient females to determine the full-term developmental potential. On the other hand, in order to compare the susceptibility to DEHP exposure between spermatozoa and preimplanted embryos, naturally fertilized oocytes and recovered 2-cell embryos were cultured in DEHP-supplemented medium to monitor the stage in which DEHP took effect.

Despite the increasing concern with reproductive impacts associated to DEHP and their relevant gene-environment interactions, there remains a paucity of studies that focuses on genomic changes after DEHP exposure. It was reported that DEHP exerted organ-specific mutagenicity [13], and DEHP administration to pregnant mice altered embryonic gene expression critical for fetal development, both in *in vivo* and *in vitro* experiments [14], [15], [16]. Thus, potential genetic aberrations contribute to DEHP hazard, have attracted our eyes. Transgenic animal model-Big Blue® mice were adopted in the study. As a mutation detecting system, genome of Big Blue® mouse is stably inserted with a *λ* shuttle vector containing bacterial *lacI* gene, which encodes a transcription inhibitor for *lacZ* [17]. Any mutation in *lacI* will start up transcription of its adjacent *lacZ* gene, as the translational product of *lacZ*, activity of*β*-galactosidase could be displayed in bacteria with a color assay in presence of 5-bromo-4-chloro-3-indolyl-*β*-d-galactopyranoside (X-gal). Thus potential genetic aberrations resulted from DEHP exposure would be manifested as an increase in mutation frequency (MF), facilitating the comparison of the frequency of rare point mutations specifically. Animal experiments [18] and human epidemiological studies [19] have indicated the predominance of adverse effects of DEHP in males targeting at testes. If these effects occur in genome of testes, the target organ could produce enough plaque forming units (PFUs) for statistics. In this study, we used Big Blue® mouse, with four-week *in vivo* DEHP exposure through intraperitoneal injection (500 mg/kg/day), to determine any relative difference in the frequency of *de novo* point mutations in testicular genome.

## Materials and Methods

### Ethical statement

This study was carried out in full compliance with Guide for the Care and Use of Laboratory Animals. The protocol was approved by the Committee on the Ethics of Animal Experiments of Shanghai Institute of Planned Parenthood Research.

### Experimental design

DEHP (Sigma-Aldrich, St. Louis, MO) was dissolved in DMSO (Sigma-Aldrich, St. Louis, MO). DEHP and DMSO were used at the final concentration of 1 μg/ml and 0.1%, respectively. Considering the potential effect of the solvent, the vehicle control (DMSO) was used as control for all *in vitro* experiments. To investigate the effect of DEHP on fertilization and embryonic development, spermatozoa were placed in DEHP-added HTF (1 μg/ml) medium for 30 min, washed by DEHP-free HTF and then incubated in fresh HTF medium until capacitation finished. Control spermatozoa were processed with the same procedure except the exposure of DEHP. Part of 2-cell stage embryos were randomly selected to transfer into recipient mice to determine the full term development. To study the effect of DEHP on the development of embryos that skipped the stage of fertilization or the first cleavage, zygotes with 2 pronuclei as well as 2-cell stage embryos by natural insemination were cultured in DEHP-added KSOM medium (1 μg/ml) to observe how toxicant worked at subsequent developmental stage.

### Animals

8–10 weeks old female B6D2F_1_ (C57BL/6×DBA/2) strain mice were used as oocyte donors, and 10–12 weeks old male B6D2F_1_ mice were used as semen donors. 8–12 weeks old wild-type ICR (CD-1) strain females previously mated with vasectomized males of the same strain were served as pseudopregnant recipients, and ICR females who experienced naturally conception and production at the same period were selected as nursing mothers. Big Blue® mice (Taconic Laboratories, Germantown, NY) were employed to perform the mutagenesis assay. All mice were housed under controlled light conditions (12 h light: 12 h dark) in the Laboratory Animal Services Facility and were fed a standard mouse diet and water ad libitum.

### Collection of oocytes and embryos

Mature female mice were superovulated with 10 IU of pregnant mare serum gonadotropin (PMSG) and 5 IU of human chorionic gonadotropin (HCG) at 48 h intervals. 14–16 h after HCG administration, cumulus oocyte complexes (COCs) were collected from the removed oviducts and dispersed by incubation in Hepes-buffered CZB medium (HCZB) and then maintained in human tubal fluid (HTF, Sage In-Vitro Fertilization, Trumbull, CT) medium supplemented with 10% human serum albumin (HSA, Vitrolife, Gothenburg, SE) at 37°C in an atmosphere of 5% CO_2_ in air until use.

With regard to the recovery of the naturally fertilized zygotes and embryos, several female mice were mated with males and examined 12–18 h after HCG injection for the presence of copulation plugs. Fertilized oocytes and 2-cell embryos were recovered by flushing the oviducts 24 h and 40 h later after HCG injection, respectively. The cumulus of oocytes were dispersed with 0.1% hyaluronidase (Sigma-Aldrich, St. Louis, MO) and washed in several changes of HCZB medium. Fertilized oocytes (identified by the presence of a second polar body and two pronuclei) as well as 2-cell embryos were then placed in KSOM medium (Millipore-Chemicon, Billerica, MA) overlaid with mineral oil, which had been equilibrated previously and cultured in a humidified atmosphere of 5% CO_2_ in air at 37°C.

### *In vitro* fertilization procedure and embryo transfer

IVF procedure was performed as previously described [20]. HTF medium overlaid with mineral oil was equilibrated in a 37°C, 5% CO_2_ incubator one day before experiment. Next day, cauda epididymides were collected from adult male mice. A dense sperm mass was squeezed out and then incubated in HTF medium for 1–2 h at 37°C to develop their fertilization potential (capacitation). A small volume of capacitated sperm suspension was added to a drop of 200 μl HTF medium containing freshly ovulated oocytes to achieve a final sperm concentration of 10^6^/ml. Four to six hours later, fertilized oocytes at pronuclear stage were washed and cultured in KSOM until embryos developed into morula/blastocyst (day 4). Oocytes were observed for male and female pronucleus formation (fertilization) at 6 h after the initiation of culture, and the number of 2-cell embryos, 4-cell embryos, morulae and blastocysts after 24 h, 48 h, 72 h and 96 h in culture, respectively. In terms of embryo transfer, 2-cell stage embryos at 24 h after insemination were transferred into the oviducts of 1-day-delayed recipient females rendered pseudopregnant by mating to vasectomized males the night preceding embryo transfer. 3–5 embryos were transferred into one oviduct, the day of transfer was considered day 0.5 of pregnancy. At day 18.5 dpc, the offspring were delivered by cesarean section and allowed to mature, the body and placental weights and sex were recorded.

### Mutagenesis assay of testes by *in vivo* exposure of DEHP

Male Big Blue® mice were intraperitoneally injected at 3 weeks of age with DEHP at 500 mg/kg/day, over a four-week period, as the dosage was referred to in former literatures [21], [22]. Control animals were treated with corn oil only. Animals were sacrificed at 1 week post-DEHP treatment while testes and cauda epididymides were acquired for mutagenesis assay and sperm chromatin dispersion (SCD) assay, respectively. High-molecular-weight genomic DNA was isolated from testes using the RecoverEase DNA isolation kit (Stratagene, La Jolla, CA) according to the manufacturer's recommendations. Mutagenesis assay was conducted as previously described with minor revision [23], [24]. In short, the *λ* shuttle vector containing the *lacI* target was recovered from genomic DNA using Transpack packaging extract kit (Stratagene, La Jolla, CA). The packaged phages were pre-adsorbed to *Escherichia coli* SCS-8 cells for 30 min at 37°C, mixed with pre-warmed NZY top agar containing 1.5 mg/ml X-gal and poured into 25×25 cm NZY agar assay tray. The plates were incubated overnight at 37°C and scored for mutant plaques. Mutant plaques were identified by their blue color, counted, cored, and replated on fresh X-gal/NZY plates to confirm and purify phage displaying the *lacI* mutant phenotype. Packaging and plating were repeated for the DNA samples until at least 300,000 PFUs were scored for each data point. Final MF was determined by dividing the number of confirmed, independent mutant plaques by the total number of PFUs. Generally, DNA extraction, *λ* packaging, and *lacI* mutant plaques plating were carried out in a “blocked” manner so as to minimize bias from day-to-day variations in experimental procedures.

### Sperm chromatin dispersion (SCD) assay

The *in vitro* DEHP-exposed spermatozoa were collected after 30 min DEHP-added HTF incubation while the *in vivo* samples were squeezed out from cauda epididymides of DEHP-exposed Big Blue® mice. Generally, SCD assay was developed as the Halosperm® kit (INDAS Laboratories, Madrid, Spain) instructed. An aliquot of each semen sample was diluted to 5–10 million/ml in PBS. The unfixed suspensions were mixed with 1% low-melting-point aqueous agarose (to obtain a 0.7% final agarose concentration) at 37°C. Aliquots of 20 μl mixture were pipetted onto a glass slide precoated with 0.65% standard agarose, covered with a coverslip (22×22 mm), and left to solidify at 4°C for 5 min. Then coverslips were carefully removed and slides immediately incubated with freshly prepared acid denaturation solution for 7 min (RT) in the dark to generate restricted single-stranded DNA (ssDNA) motifs from DNA breaks. The denaturation was then stopped, followed by incubation with lysing solution for 23 min (RT). Slides were thoroughly washed in deionized water for 5 min, dehydrated in sequential 70%, 90%, and 100% ethanol baths (2 min each) and air dried. Afterwards, cells were stained with modified Wright-Giemsa stain (Sigma-Aldrich, St. Louis, MO) for bright-field microscopy and a minimum of 400 spermatozoa per sample were evaluated under the ×40 objective of the light microscope. After staining, four SCD patterns were established: sperm heads with (i) large size halos, whose halo width was similar or larger than the minor diameter of the core, (ii) medium size halos, whose halo size was between those with large and with small halo. (iii) small size halos, whose halo width was similar or smaller than one third of the minor diameter of the core and (iv) without a halo or degraded sperm cells, the latter ones weakly or irregularly stained. The spermatozoa without DNA damage showed nucleoids with large- or medium-sized halos of spreading DNA loops whereas those with fragmented DNA appeared with a small or no halo. Finally, the percentage of sperm (iii) and (iv) was considered as DNA fragmentation index (DFI) for each semen sample.

### Statistical Analysis

*In vitro* developmental outcomes and SCD results were evaluated by SPSS software (Version 15.0; SPSS Inc., Chicago, IL, USA) for significance, using *χ^2^* tests and one-way analysis of variance (ANOVA), respectively. Mutation frequency data were analyzed by using SAS PROC GENMOD software (version 9.1; SAS Institute, Cary, NC). A poisson regression model with parameter estimates obtained by the method of maximum likelihood. Statistical tests of differences used the likelihood ratio test. Results were considered statistically significant at *P*<0.05.

## Results

### Inﬂuence of DEHP exposure on sperm fertilization and embryonic development

After incubation in DEHP-added HTF (1 μg/ml) medium for 30 min, followed by washing steps, the motility and viability of spermatozoa were not significantly changed compared to control spermatozoa (Data not shown). Spermatozoa in both groups were adjusted to the concerntration of 1×10^6^ and then applied to IVF procedure. As shown in Table 1, DEHP-treated spermatozoa retained their fertilization potential while statistically decrease existed in pronucleus formation compared to non-treated controls (63.2% vs. 74.6%, *P*<0.05). What's more, the first cleavage rate and morula/blastocyst formation rate in DEHP-treated group (92.5% and 75.1%) were also reduced strikingly compared to those of the control group (97.0% and 85.2%), respectively. With regard to the full term development, 2-cell stage embryos that derived from DEHP-treated spermatozoa were transferred into recipient mice. As shown in Table 2, the reduction of the rate of implanted embryos reached no significance (27.2% and 18.3% for control and treated spermatozoa, respectively), whereas there was an approximately 15% decrease in birth rate of live pups of treated spermatozoa (26.1% vs. 11.7%, *P*<0.05), demonstrating a remarkable adverse outcome because of the effect of DEHP. Although fewer offspring resulted from treated spermatozoa than fresh controls, their body and placental weights were still within normal range, and the mice grew to adulthood. Three male and four female mice derived from DEHP treated spermatozoa kept normal and health phenotype, with no evident abnormalities found in reproductive system. The above results showed that the reduction of birth rate of live pups was in accordance with the tendency of decreased embryonic development *in vitro*. Considering that the potential hazards of toxicants has been suggested differ according to different developmental and differentiation state of target cells and tissues [25], and since the detrimental effect of direct low-level DEHP exposure on spermatozoa had been observed, we further wanted to know whether fertilized oocytes and 2-cell embryos were also targets of DEHP. In *in vitro* culture experiment where embryos derived from natural insemination, both fertilized oocytes and 2-cell embryos were divided into two groups at random: DEHP-free KSOM and DEHP-added KSOM. Under the concentration of DEHP here adopted, all oocytes and embryos survived the exposure, with no evident changes in cell morphology. With regard to naturally fertilized oocytes, there were significant differences in the rate of first cleavage and morula/blastocyst formation between DEHP-free KSOM and DEHP-added KSOM groups as shown in Table 3. DEHP effectively blocked fertilized oocytes from reaching 2-cell embryo stage, and subsequently decreased the formation of morula and blastocyst (*P*<0.05). In contrast, the 2-cell embryos which completed their first cleavage process *in vivo* appeared resistant to exogenous DEHP in culture medium and showed no evident adverse effect on development progression (*P>*0.05).

**Table 1:** The effect of DEHP upon fertilization and *in vitro* embryonic development by IVF procedure.

Treatment	No. of oocytes	No. with pronuclei formation (%) a	No. of 2-cell embryos (%) b	No. of morulae/blastocysts (%) b
Control spermatozoa	362	270 (74.6)	262 (97.0)	230 (85.2)
DEHP-exposed spermatozoa	318	201 (63.2)*	186 (92.5)*	151 (75.1)*

a Compared with oocytes.

b Compared with pronuclear embryos.

* *P*<0.05 compared with control group.

**Table 2:** Production of offspring derived from DEHP-treated spermatozoa.

Group	No. of 2-cell embryos transferred	No. of embryos implanted	No. of live pups (%)	Survive after 20 weeks (%)	Weight (g) ± SD	
					Pups	Placenta
control spermatozoa derived	92	25(27.2)	24(26.1)	24(100)	1.56±0.21	0.15±0.04
DEHP-exposed spermatozoa derived	60	11(18.3)	7(11.7)*	7(100)	1.51±0.27	0.14±0.03

* *P*<0.05 compared with control group.

**Table 3:** The effect of DEHP upon the development of naturally fertilized oocytes and 2-cell embryos.

Category	No. of fertilized oocytes	No. of 2-cell embryos (%)	No. of morulae (%)	No. of blastocysts (%)
Fertilized oocytes with DEHP-free medium	278	269(96.8)	251(90.3)	248(89.2)
Fertilized oocytes with DEHP-added medium	483	420(87.0)*	366(75.8)*	339(70.2)*
2-cell embryos with DEHP-free medium		253	246(97.2)	244(96.4)
2-cell embryos with DEHP-added medium		310	299(96.5)	290(93.5)

* *P*<0.05 compared with DEHP-free control.

### Inﬂuence of DEHP exposure on genomic mutation frequency in testes

The mutation frequencies observed in control and DEHP-treated animals were summarized in Table 4. The average spontaneous MF in testes of control mice was (0.97±0.11)×10^−5^, which was similar to MF previously reported in control mice [26]. When treated over four weeks, an approximately 3-fold increase in MF (2.71±0.20)×10^−5^ was observed in the testes of mice exposed to DEHP, which was notably elevated compared with that of control (*P*<0.01).

**Table 4:** Mutation frequency in the testes of Big Blue® mice following 4-week DEHP exposure. SD  =  standard deviation.

DEHP dose	No. of mice	Total No. of PFUs	No. of mutant plaques	MF ± SD (×10^−5^)	*P*-value
0mg	7	2533600	24	0.97±0.11	<0.01
500mg	7	3090000	82	2.71±0.20	

### Inﬂuence of DEHP exposure on sperm DNA integrity

We next investigated whether DEHP-induced reproductive hazards was associated with sperm DNA damage using SCD assay. Both the spermatozoa exposed to DEHP *in vitro* and *in vivo* displayed an increase in average percentage of DNA fragmented spermatozoa after treatment compared with that of control groups, but did not reach a significant difference (*P*>0.05), as shown in Table 5.

**Table 5:** The SCD results of DEHP exposure upon sperm DNA integrity.

Treatment	Control (DFI, %)	DEHP-exposed (DFI, %)	*P*-value
*In vitro*	7.30±1.82	7.67±1.54	>0.05
*In vivo*	7.37±1.23	9.17±2.02	>0.05

## Discussion

Increasing public concern over environmental phthalate distribution persisted since phthalates cause human reproductive abnormalities, wherein DEHP might be one of the most environmentally abundant phthalates. DEHP is loosely chemically bonded to plastic, therefore it can leach out of the lining of plastic packages, cans and baby bottles, and pipe walls then readily into blood or other lipid-containing solutions in contact with the plastic. Human body is reported to expose to concentrations of 30 µg/day of DEHP, and it has been detected in human body samples, such as serum, urine, amniotic ﬂuid of pregnant women, breast milk and even in semen [27], [28], [29]. Despite widespread general population exposure to DEHP, limited data exist towards the potential general population effects of phthalate exposure on spermatozoa or embryos. The present study reported that direct DEHP exposure would diminish fertilization capacity and embryonic development, extrapolating experimental data favoring public reproductive hazard of DEHP. Former animal testing of phthalates were always conducted at doses far higher than those present in the ambient human environment, yet failed to imply human health under nonlinear dose response as in cases of other environmental chemicals [30]. Therefore, considering that the Environmental Protection Agency (EPA) has established a DEHP safety concentration limit in drinking water at 6 ppb (μg/l) [31] and the real endosomatic level in human subjects [27], [32], wherein infertile men in India who present DEHP levels of up to 0.77±1.20 µg/mL in semen have sperm abnormalities [32], we adopted very low-dose DEHP (1 μg/ml) in *in vitro* experimental design in order to closely mimic the effects of DEHP on spermatozoa and relate animal experiments to human health. In this paper, the low level of DEHP was associated with modest percentage decreases in the rate of pronuclei and morulae/blastocysts formation (63.2% and 75.1% for treated spermatozoa, and 74.6% and 85.2% for control ones, respectively), indicating a diminished developmental potential of DEHP-contaminated spermatozoa. This degree of decrease may have a minimal biological impact on the fertility for a given individual, however, on the population level, the public health significance of a shift in the fertilization capacity and developmental potential of embryo would be large. Although DEHP exposure can reduce the function of spermatozoa, part of oocytes fertilized with these spermatozoa still retained their full-term developmental potential after transferred. The decreased proportion of transferred embryos implanted in uterus and the significant reduction of live birth were in accordance with the tendency of decreased embryonic development *in vitro*, as we expected. In the presence of low-level DEHP in culture medium, naturally fertilized oocytes were remarkably affected from the first cleavage stage to the blastocyst stage whereas recovered 2-cell embryos remained uninfluenced throughout all stages, suggesting DEHP exposures at particular developmental stages of embryogenesis presented differential responses, and the working-points may target mainly at fertilization and the first cleavage process. Therefore we should pay particular attention and take measures to prevent DEHP exposure during these stages, not only in assisted reproductive technology (ART) treatments but also natural conception. At higher concentration, consistent with our results, DEHP exhibited embryotoxic potential, using an *in vitro* battery system containing an entire embryo cultured for 48 h, 9.5 days after gestation [33]. Taken together, the low-level DEHP appeared to have harmful effects on mouse spermatozoa functions and embryogenesis-at least during fertilization and 2-cell embryo formation stages, and if protected from DEHP at the stages of fertilization and 2-cell formation, the embryonic development could proceed successfully.

In line with our opinion that DEHP may interfere with the reproduction ability, a study by analyzing the concentration of phthalates metabolites in spot urine samples, indicated that a significantly higher excretion of these metabolites was found in couples who seek for ART help than control parents of one or more children [34]. Epidemiological studies performed in infertility clinics, with exposure levels within the range of the body burden of the general population, revealed that semen parameters might be affected by environmental exposure [12], [35]. These facts confirmed the hypothesis that exposure to phthalates, from various sources, can affect human fertility. On the other hand, though sperm function was diminished but not abolished in our study, it could be speculated that while infertile males suffered DEHP contamination, IVF could still qualified as a sensible and feasible approach in ART therapy. Even though it is still unclear how DEHP could result in reproductive damages with direct exposure on sperm or embryo cell, this current finding indicated that the detrimental effects of DEHP may occur at very low dose levels, is of importance, contributing to a new insight toward the reproductive toxicity and disruptive function upon mammalian fertility of DEHP.

One of DEHP's primary application fields is the medical industry, which may make people expose to more DEHP during medical treatment. During ART treatment such as IVF is conducted, DEHP may leaches out of plastic containers such as semen collection cup, centrifugal tube and culture dish into the culture medium. A very recent study reported the DEHP contamination in IVF media, sperm washing media and protein source at a concentration of <10–114, <2.0–263, and <10–982 ng/mL, respectively [36]. Although our study did not show any influence from DEHP existing in laboratory environment, this kind of iatrogenic exposure must be paid attention in infertility clinics. Meanwhile, our results showed that under the same concentration of DEHP, IVF assay is more sensitive than detection of sperm parameter to determine the effect of DEHP exposure on spermatozoa, suggesting that IVF could be applied when laboratory quality control and evaluation is conducted in those institutes for infertility treatment or research.

Although DEHP has been reported to be negative in many non-mammalian *in vitro* mutation assays, most studies were performed under conditions of concurrent cytotoxicity, precipitation, or irrelevant metabolic activation [11]. In human tissues and cells, it was reported that a similar DEHP concentration range induced both mutagenic and non-mutagenic effects [11]. There were two *in vivo* mammalian mutation assays for DEHP exposure available. One study showed that after 21 days exposure (6 doses of 2333 mg/kg DEHP), there was a significantly elevated MF in both male and female liver DNA (∼40%), but not in kidney and spleen [13]. Another assay that used 5 female guanine phosphoribosyltransferase delta transgenic rats, *in vivo* mutagenicity and mutation spectra results after 13-weeks of DEHP treatment (187 mg/kg/day) were proved to be negative [37]. In line with the notion that DEHP might exert organ-specific genotoxic effect [13], in this study, the data obtained using Big Blue® mice over a 4-week exposure unambiguously demonstrated that spontaneous MF increased in adult testes tissues after DEHP exposure. Given the Big Blue® assay was considered as a versatile and sensitive *in vivo* mutational model, we could speculate that this 3-fold increase in MF pose considerable genetic risks once these mutation points occurred at key position of important genes that regulate spermatogenesis in testes. And increased germ-line DNA mutation frequencies may cause population-level changes in genetic composition and disease [38], elevated MF in rodent testes would imply long-term risks relevant to the development of genetic diseases in subsequent generations. In conclusion, this *in vivo* experiment was important, as preliminaries or components of studies on germ line mutagenicity, paving the way for human genetic risk assessment and genetic hazard prediction of DEHP, though potential health effects warranted extensive further investigation. When it comes to the correlation between DEHP exposure and sperm DNA integrity, it still remained controversial. One study reported that semen DEHP levels strongly associated with morphologic abnormality and DNA fragmentation index in the general Indian population [32]. Some others observed that there were no significant relationships between urinary sperm DNA damage and MEHP [39], [40]. Our study showed that neither low-level *in vitro* nor high-level *in vivo* DEHP exposure would result in significant sperm DNA damage. The *in vitro* data demonstrated that sperm DNA integrity was resistant to short-term exposure of low-level DEHP and developmental abnormality could occur in the absence of detectable sperm DNA lesion. Meanwhile, it was notable that though not significant, DFI increased to a certain degree after long-term exposure *in vivo*, indicative of potential damage to sperm DNA integrity. It was possible that varied dose levels and exposure routes of DEHP; different detection sensitivity in DNA damage quantification; different working concentrations due to administration routes, collectively contributed to the controversy of association between DEHP and sperm DNA damage.

How DEHP exerts hazards remains unclear, cell transformation, intercellular communication, and apoptosis/proliferation gene expression changes gene changes, modification of enzyme activity and DNA methylation are possibly involved in the mechanisms [11]. It has been established that DEHP disrupts oxidative balance associated with production of a strongly oxidative damaging milieu [41], [42], increased the production of reactive oxygen species (ROS) and decreased production of protective antioxidants, as evidenced by significant decreases in glutathione peroxidase 1 (GPx1) (∼20%) and superoxide dismutase (SOD) (∼30%) activities and glutathione (GSH) levels (∼20%) in DEHP treatment (1000 mg/kg) rats [43], and might ultimately leading to carcinogenesis [44], [45]. There are several other lines of evidence relating DEHP to reproduction, such as altering the expression of several key genes in embryonic zinc homeostasis [46], inhibited equine oocyte maturation [47] and blocked mouse follicle growth through an oxidative stress pathway [48]. Partial mechanisms were also revealed by *in vivo* exposure of DEHP in *Danio rerio*, where a significant reduction of fecundity was observed. In female *Danio rerio*, DEHP affected signals involved in oocyte growth, maturation and ovulation, thus deeply impaired ovarian functions with serious consequences on embryo production [49]. While in male *Danio rerio*, high concentrations of DEHP disrupted spermatogenesis in adult *Danio rerio* via meiotic progression inhibition and consequently decreased their ability to fertilize oocytes [50]. Since a significant genetic similarity exists between *Danio rerio* and human, the harmful effects observed at oocyte and sperm level may stimulate further molecular studies on humans.

In conclusion, the present study unveiled the hazardous effects of direct exposure of low-level DEHP on fertilization ability and embryonic development, both *in vitro* culture process of preimplanted embryos and full-term development until live birth presented significant impairment, wherein spermatozoa and fertilized ooctyes before the first cleavage seemed more susceptible to DEHP exposure. Meanwhile, the mutagenesis assay of Big Blue® mice proved that DEHP posed mutagenic risks in the reproductive organ-at least in testes. Therefore, considering the extensive use of plastic products for medical purpose and the possibility that DEHP may exert adverse effect at very low level, we should reduce both environmental and iatrogenic exposure of DEHP at all possible to protect our reproductive capacity.
